# Breast density analysis of digital breast tomosynthesis

**DOI:** 10.1038/s41598-023-45402-x

**Published:** 2023-10-31

**Authors:** John Heine, Erin E. E. Fowler, R. Jared Weinfurtner, Emma Hume, Shelley S. Tworoger

**Affiliations:** 1 Cancer Epidemiology Department, Moffitt Cancer Center and Research Institute, 12902 Bruce B. Downs Blvd, Tampa, FL 33612 USA; 2 Diagnostic Imaging and Interventional Radiology, Moffitt Cancer Center and Research Institute, 12902 Bruce B. Downs Blvd, Tampa, FL 33612 USA

**Keywords:** Breast cancer, Image processing, Risk factors, Statistics

## Abstract

Mammography shifted to digital breast tomosynthesis (DBT) in the US. An automated percentage of breast density (PD) technique designed for two-dimensional (2D) applications was evaluated with DBT using several breast cancer risk prediction measures: normalized-volumetric; dense volume; applied to the volume slices and averaged (slice-mean); and applied to synthetic 2D images. Volumetric measures were derived theoretically. PD was modeled as a function of compressed breast thickness (CBT). The mean and standard deviation of the pixel values were investigated. A matched case–control (CC) study (n = 426 pairs) was evaluated. Odd ratios (ORs) were estimated with 95% confidence intervals. ORs were significant for PD: identical for volumetric and slice-mean measures [OR = 1.43 (1.18, 1.72)] and [OR = 1.44 (1.18, 1.75)] for synthetic images. A 2nd degree polynomial (concave-down) was used to model PD as a function of CBT: location of the maximum PD value was similar across CCs, occurring at 0.41 × CBT, and PD was significant [OR = 1.47 (1.21, 1.78)]. The means from the volume and synthetic images were also significant [ORs ~ 1.31 (1.09, 1.57)]. An alternative standardized 2D synthetic image was constructed, where each pixel value represents the percentage of breast density above its location. Several measures were significant and an alternative method for constructing a standardized 2D synthetic image was produced.

## Introduction

Breast density is a significant and well accepted breast cancer risk factor assessed from mammograms^1–4^. Areas of increased breast density (i.e., the degree of bright tissue) correspond to tissue with greater x-ray attenuation, as observed in mammograms used for clinical purposes. Breast density is one factor among others that could be considered in the development of breast cancer risk prediction models for clinical purposes. These models could be used for developing personalized healthcare strategies, such as setting risk-modulated screening intervals or imaging modality choice, providing the accuracy permits^5,6^.

In the current clinical environment, there are several breast cancer risk models used for specific purposes^4,7^ for example: the Gail model^8^ is used to advise on chemoprevention for reducing risk; the Tyrer-Cuzick^9^, BRCAPRO^10,11^ and Claus^12^ models are useful for determining if supplemental imaging with magnetic resonance might be beneficial^4^. The Breast Cancer Screening Consortium (BCSC) model^13,14^ may be useful for determining if women with dense breasts require supplemental screening^13^. These models do not use the same set of risk factors and are useful for different subpopulations^15^. For example, the BRACPRO model is used for predicting the risk of carrying a genetic mutation, and the Claus model is based on a family history of breast cancer. It is worth noting the Tyrer-Cuzick and BCSC models use breast density. Recently, the American College of Radiology (ACR) provided recommendations for supplemental imaging based on risk in conjunction with breast density^16^. It is also worth noting at present for screening in many high-income countries, risk is based primarily on age^17^. Risk prediction methods that use some type of image derived information (simple modeling through deep learning) show that texture may be an important factor, yet it is not commonly used in practice^4,18,19^.

There are many methods under investigation for measuring both breast density and more generally texture^19–22^. The percentage of breast density measure (PD) has been studied for many years and has repeatedly shown to be significantly associated with breast cancer risk^23,24^. PD requires determining a threshold in a 2D mammogram. All pixels above this threshold are labeled as dense or otherwise labeled as non-dense creating a binary image. The final measure is derived from normalizing the dense area by the total breast area, presented as percentage. The ACR Breast Imaging Reporting & Data System (BI-RADS) four state ordinal breast composition classification^25^ has also been used as a risk measure and shows consistent risk prediction capability across studies and time^1^. These measures capture the volumetric tissue characteristics projected onto a plane. Newly derived image markers are often compared to PD as it has been considered as the de facto benchmark standard. For example, recent work shows that PD produces a risk measure marginally superior to a commercially available volumetric breast density product when studying 2D full field digital mammography (FFDM) images^26^.

Breast screening recently has largely transitioned from FFDM to digital breast tomosynthesis (DBT) in the US. DBT provides a *three-dimensional* (3D) rendering of the breast via stacked 2D images (slices) derived from 2D projection images acquired over a limited angular range. Clinical DBT images result from heavy processing. There is little published work with measures derived from clinical DBT images for risk factor purposes at this time^19,27^. It is reasonable to assume that a more precise measure of breast density would result from analyzing volumetric images in comparison with conventional 2D mammograms. Accordingly, recent work in comparing an automated volumetric measure from DBT with measures applied to 2D mammograms shows improvements in risk prediction capability^28^. Moreover, recent modeling using DBT data, incorporating various images features, illustrates it is possible to guide image care^18^.

We previously developed an automated PD approach (PD_a,_) that was evaluated in studies with digitized film and FFDM images^29–31^. In this report, we will apply this method to DBT data^32^ and evaluate its merits for risk prediction. A matched case–control study was investigated with women that have DBT volume images (2D slices) and synthetic 2D mammograms (referred to C-View images in the mammography technology applicable to this report). There are three main study objectives. First, we investigated an algorithm modification used recently when studying relatively low-resolution digitized film mammograms^31^ and evaluated it with DBT without training or additional testing (i.e., a blind evaluation). Secondly, we derived different breast density measures from DBT volume data and compared these measures with PD determined from the C-View images in their risk prediction capabilities. Thirdly, PD was modeled as a function of compressed breast thickness and investigated.

## Materials and methods

### Study data

Study data was obtained from women participating in one of two studies collected under the same Institutional Review Board (IRB) of the University of South Florida, Tampa, FL (08/13/2007) selection protocol. Data was collected both retrospectively on a waiver for informed consent and prospectively with informed consent, both approved by the IRB referenced above. We investigated a matched case–control study (n = 426 pairs). Selection criteria and study population were described previously^33,34^. Briefly, cases were women with first time pathologically verified unilateral breast cancer from two sources: (1) women attending the breast clinics at Moffitt Cancer Center (MCC) diagnosed with breast cancer, and (2) attendees of surrounding area clinics sent to MCC for either breast cancer treatment or diagnostic workup and found to have breast cancer. Controls were attendees of this center without history of breast cancer, verified by a two-year follow-up. Controls were individually matched to cases on these criteria: age (± 2 years), hormone replacement therapy (HRT) usage and current duration (never users or not current users in the prior 2 years, current user ± 2 years duration), screening history (any prior screening with time since last screening < 30 months, any prior screening > 30 months before baseline, no prior screening), and mammography unit (described below). Cranial caudal orientation mammograms were used for this study to reduce pectoral interference in the automated analyses. The unaffected breast image(s) were used for cases (all study images were acquired before treatment) and the matching lateral breast image for controls. Population characteristics are shown in Table 1.

**Table 1:** Population characteristics: participant numbers (n) are broken down by case–control status and totals.

Measure or characteristic	*p* value	Case n	Case mean, SD, or relative frequency	Control n	Control mean, SD, or relative frequency	Total n	Total mean, SD, or relative frequency
Age*	0.7787	426	58.2 (11.6)	426	58.2 (11.6)	852	58.2 (11.6)
BMI	< 0.0001	426	29.6 (6.9)	424	27.7 (6.3)	850	28.7 (6.7)
Screening group*	N/A						
Group 1		262	61.50%	262	61.50%	524	61.50%
Group 2		87	20.42%	87	20.42%	174	20.42%
Group 3		77	18.08%	77	18.08%	154	18.08%
HRT usage*	N/A						
Current		36	8.45%	36	8.45%	72	8.45%
Not currently		390	91.55%	390	91.55%	780	91.55%
Race							
Caucasian	0.5394	340	79.81%	348	81.69%	688	80.75%
African American	1.0000	47	11.03%	46	10.80%	93	10.92%
Asian	0.5716	16	3.76%	12	2.82%	28	3.28%
Other	1.0000	12	2.82%	12	2.82%	24	2.82%
More than one	0.0703	7	1.64%	1	0.23%	8	0.94%
Unknown	0.5488	4	0.94%	7	1.64%	11	1.29%
Ethnicity	< 0.0001						
Non-Hispanic		369	86.62%	323	75.82%	692	81.22%
Hispanic		57	13.38%	98	23.00%	155	18.19%
Unknown		0	0.00%	5	1.17%	5	0.59%
Menopausal status							
Premenopausal	1.0000	114	26.70%	111	26.06%	225	26.41%
Postmenopausal	0.7877	311	73.13%	311	73.00%	622	73.00%
Unknown	0.3750	1	0.17%	4	0.94%	5	0.59%

Where applicable, distribution means and standard deviations (SDs) are provided. Matching factors are indicated by asterisks.

Mammograms were acquired with Hologic Dimensions DBT units (Hologic, Inc., Bedford, MA): *volume* images (in-plane 100 μm average pixel spacing, 1 mm slice thickness, with 10-bit pixel dynamic range); and synthetic (i.e., C-View as named by this manufacturer) 2D images (100 μm average pixel spacing with 10-bit pixel dynamic range). The in-plane pixel spacing varies for DBT *volume* slices and C-View 2D images simultaneously and in tandem for each acquisition, ranging from approximately 85 μm to 106 μm (about 100 μm average) for our data. The number of slices in each DBT volume image is approximately one slice per mm of compressed breast thickness. It is important to note that DBT slices and the C-View images result from heavy processing unlike raw (*for processing*) FFDM images. Because this work involves creating an alternative 2D synthetic image, we use the manufacture’s C-View nomenclature when referring to the respective synthetic 2D images used for clinical purposes and synthetic when referring to the experimental images produced in this report to avoid confusion.

### Automated PD detection algorithm

Our automated PD detection mechanism (referred to here as, PD_a_) has been under investigation for many years, necessitated by both changing mammography technologies and implementing algorithm improvements based on our image-understanding^29,30,35–37^. The methodology operates by analyzing signal dependent noise locally in 2D images^29^. We refer to this approach as a *detection algorithm* due to the way it makes systematic (probabilistic) local decisions to identify dense tissue. PD_a_ is a two-stage detection algorithm that first determines a global reference variance for adipose tissue (over the entire breast area) in a high pass wavelet filtered version of the image. In the first detection stage, this reference variance is used for making statistical comparisons with local variances determined from a n × n box (n = 4) scanned systematically across the breast area. Local regions that deviate significantly from this reference are labeled as dense or otherwise non-dense by default. In the second detection stage, the reference adipose variance is refined by estimating it from the non-dense areas identified in the first stage. The localized comparisons are then repeated with the refined reference resulting in the PD_a_ labeled image. Each detection stage requires a threshold defined by a significance level selected a priori from a Chi-square distribution.

In previous work, we noted applying a non-linear transform to the images before performing the density detection process had a beneficial impact on the algorithm's output^29,36,38^. Through experimentation with 2D *for presentation* images from General Electric Senographe 2000D (Milwaukee, WI) [180 case–control pairs] and Hologic Selenia units [320 case–control pairs], we found that a 0.1 significance level was robust for both detection stages [i.e., determined from images that were processed in some way after the acquisition process unlike raw (*for processing* images)] by first multiplying mammograms with random noise (zero-mean unit variance normally distributed) before applying the high pass wavelet filter (i.e., the same *trick* used for the low-resolution film analysis). This extra step appears to boost the signal dependent noise signal in the high pass wavelet image; understanding this mechanism is a separate investigation underway. DBT data has undergone heavy processing and has relatively greater pixel spacing (i.e., reduced resolution) than Hologic 2D FFDM images used in this study. Therefore, we applied this noise multiplication modification to all DBT images investigated in this report and performed the detection with the preset detection parameters. As part of *this blind investigation* (verification), detection thresholds (both stages) were set using a significance level = 0.1 with 15 degrees of freedom (i.e., n^2^ − 1) without analyzing DBT data with the modified PD_a_ methodology a priori. Because there is difficulty in determining ground truth for breast density, we will use the statistical significance of the OR findings as the objective endpoint benchmarks.

### Breast density measurement modeling with DBT

Multiple breast density measures can be derived from PD measurements when applied to DBT images. At the local region within a slice (or any image), we assume that the density detection process is a surrogate for approximating the probability that the region has the x-ray attenuation coefficient of dense breast tissue, and the dense tissue acceptance (detection) is based on this probability (i.e., referred to as the probability conjecture below).

For the volume derivations, we let a given DBT volume image have N slices with an isotropic thickness, t, measured in mm and pixel area given by A measured in mm^2^. The i^th^ slice has n_i_ pixels in the breast area with d_i_ pixels labeled as dense. The expression for the breast volume (BV), required in the derivations, is given by

1 $${\text{BV}} = {\text{t}} \times {\text{A }}\mathop \sum \limits_{{{\text{i}} = 1}}^{{\text{N}}} {\text{n}}_{{\text{i}}} = {\text{c }} \times {\text{N}} \times \left\langle {{\text{n}}_{{\text{i}}} } \right\rangle ,$$

where the brackets indicate the average (expectation) operator, < n_i_ > is the average number of pixels over the slice breast areas, and c = ${\text{t}} \times {\text{A}}$. As a measure, the total dense tissue volume (D_v_) within the breast volume is given by

2 $${\text{D}}_{{\text{v}}} = {\text{t}} \times {\text{A }}\mathop \sum \limits_{{{\text{i}} = 1}}^{{\text{N}}} {\text{d}}_{{\text{i}}} = {\text{c }} \times {\text{N}} \times \left\langle {{\text{d}}_{{\text{i}}} } \right\rangle ,$$

where $\left\langle {{\text{d}}_{{\text{i}}} } \right\rangle$ is average of PD taken over the N slices. Using the BV and D_v_ expressions, the volumetric PD measure (PD_vol_) is given by

3 $$ {\text{PD}}_{{{\text{vol}}}} = 100{\text{\% }} \times { }\frac{{{\text{D}}_{{\text{v}}} }}{{{\text{BV}}}} = { }100{\text{\% }} \times { }\frac{{\left\langle {{\text{d}}_{{\text{i}}} } \right\rangle }}{{\left\langle {{\text{n}}_{{\text{i}}} } \right\rangle }}. $$

Another metric can be derived by averaging PD over the N slices (PD_m_) giving

4 $${\text{PD}}_{{\text{m}}} = { }\frac{100\% }{{\text{N}}}{ } \times \mathop \sum \limits_{{{\text{i}} = 1}}^{{\text{N}}} \frac{{{\text{d}}_{{\text{i}}} }}{{{\text{n}}_{{\text{i}}} }}.$$

Equation (4) is an indication of why the normalization for PD may be important, as follows. Making the approximation that the breast area in each slice is constant for a given woman with n pixels gives

5 $${\text{PD}}_{{\text{m}}} { } \approx \frac{{100{\text{\% }}}}{{\text{N}}} \times { }\frac{{\mathop \sum \nolimits_{{{\text{i}} = 1}}^{{\text{N}}} {\text{d}}_{{\text{i}}} }}{{\text{n}}} = 100{\text{\% }} \times \frac{{{\text{N}} \times \left\langle {{\text{d}}_{{\text{i}}} } \right\rangle }}{{{\text{Nn}}}} = { }100{\text{\% }} \times \frac{{\left\langle {{\text{d}}_{{\text{i}}} } \right\rangle }}{{\left\langle {{\text{n}}_{{\text{i}}} } \right\rangle }},$$

where n = $\left\langle {{\text{n}}_{{\text{i}}} } \right\rangle$. When the breast areas are similar in each slice, PD_vol_ ~ PD_m_; this approximation will be evaluated. PD from the labeled slices can be projected (summed) onto a plane giving a (coarse) 2D image with pixel values ranging between zero and N. The summation of pixel values within this image gives the projected total (PT) expressed as

6 $${\text{PT}} = {\text{N}} \times \left\langle {{\text{d}}_{{\text{i}}} } \right\rangle .$$

If we assume the traditional PD thresholding in 2D captures the dense pixel proportion above a given location within the compressed breast, the total number of dense pixels in a 2D labeled image is approximately the normalized projected total (NPT) determined by dividing Eq. (6) by N giving

7 $${\text{NPT}} = { }\left\langle {{\text{d}}_{{\text{i}}} } \right\rangle .$$

Given there are n pixels within the breast area, the standardized 2D measure is approximated by

8 $${\text{PD}} \approx 100{\text{\% }} \times \frac{{{\text{NPT}}}}{{\text{n}}}{ } = { }100{\text{\% }} \times \frac{{\left\langle {{\text{d}}_{{\text{i}}} } \right\rangle }}{{\text{n}}},$$

which is just Eq. (3) or Eq. (5) relabeled. The projected image normalized by N and multiplied by 100% produces a standardized synthetic image [defined as s(x,y)], where each pixel represents the percentage of dense tissue volume above its location (using a parallel beam approximation); we note, these interpretations follow from the probability conjecture defined above. In these derivations, image parameters (A and t) were not relevant except for D_v_. When assessing D_v_ across women, t is constant while A will vary and must be accounted for in the metric.

We investigated odd ratios (ORs) produced when applying PD_a_ to the C-View images (PD_syn_) and when producing PD_vol_ and PD_m_. ORs produced by analyzing PD from the isolated central DBT slice were also analyzed (i.e., exploring the possibility that PD from one slice may be representative of the volume). As further comparators, we evaluated the mean and standard deviation of the pixel values within the DBT volume without PD_a_ processing, referred to as m_vol_ and v_vol_. Likewise, we used the mean from the C-view image pixel values (m_syn_) as another comparator.

To study breast density characteristics throughout the DBT volume, we used an empirically driven second-degree polynomial model expressed as

9 $${\text{PD}} = {\text{a}} + {\text{b}} \times {\text{P}} + {\text{c}} \times {\text{P}}^{2} ,$$

where PD is from each slice, P is the normalized independent slice number variable ranging from [1, 100] measured as a unitless proportion ranging from the slice at the breast support surface (P = 1) to the furthest slice from the support surface (P = 100), and (a, b, c) are parameters to be determined with curve fitting analysis. The slice distance from the breast support surface can be recovered approximately by $\frac{{\text{P}}}{100}$ × (compressed breast thickness). The convention used for increasing P follows that of the clinical volume rendering from the image header files. This normalization for distance puts the fit parameters on the same scale over all participants. We investigated parameter distributions (empirical normalized histograms) and made comparisons across case–control status. The normalized distance for the maximum PD quantity can be derived by setting the derivative of Eq. (9) to zero giving

10 $${\text{P}} = -\frac{{\text{b}}}{{2 \times {\text{c}}}},$$

which was investigated*.*

### Statistical analyses

Image measure associations with breast cancer were evaluated using conditional logistic regression while controlling for body mass index (BMI) and ethnicity. Unadjusted models are included in the tables for completeness. We used ORs with 95% confidence intervals (CIs) as the primary metric to evaluate and compare breast cancer associations between the various image measures defined above. ORs were estimated for continuous measures in per standard deviation (SD) increment. We note, an OR derived from a case–control study from a given population is often used as an approximation for relative risk for the same population, providing the disease incidence is small. Although building predictor models is not the purpose of this report, for completeness the area under the receiver operator characteristic curve (Az) was calculated with 95% CIs to summarize the discriminatory ability for each model. Both ORs and Azs are presented with CIs parenthetically. The matching design in this case–control study was implemented specifically to isolate the associations of image measures with breast cancer risk and generally precludes developing risk prediction models, which require detailed information regarding selection probabilities. Pearson correlation coefficient (R) was used to show the linear relations between select breast density measures and BMI.

### Ethics and consent to participate

All methods were carried out in accordance with relevant guidelines and regulations. All experimental procedures were approved by the Institutional Review Board (IRB) of the University of South Florida, Tampa, FL under protocol #Ame13_104715. Mammography data was collected both retrospectively on a waiver for informed consent and prospectively with informed consent both approved by the IRB referenced above.

## Results

### Population characteristics

Table 1 shows the characteristics of the case–control participants. As expected, matching variables (age, screening, HRT) were similar across case–control groups. Similarly, neither race (Caucasian, African American, or Asian) or menopausal status varied significantly by status. Menopausal status did not vary significantly as it is likely a surrogate for age. In contrast, both ethnicity (Hispanic, non-Hispanic) and BMI (larger for cases) varied significancy. The BMI finding is expected, as increased risk is associated with increased BMI^39^. The difference in ethnicity is due to the shifting demographics at our clinics.

### Illustrations and related analyses

**Figure 1:**
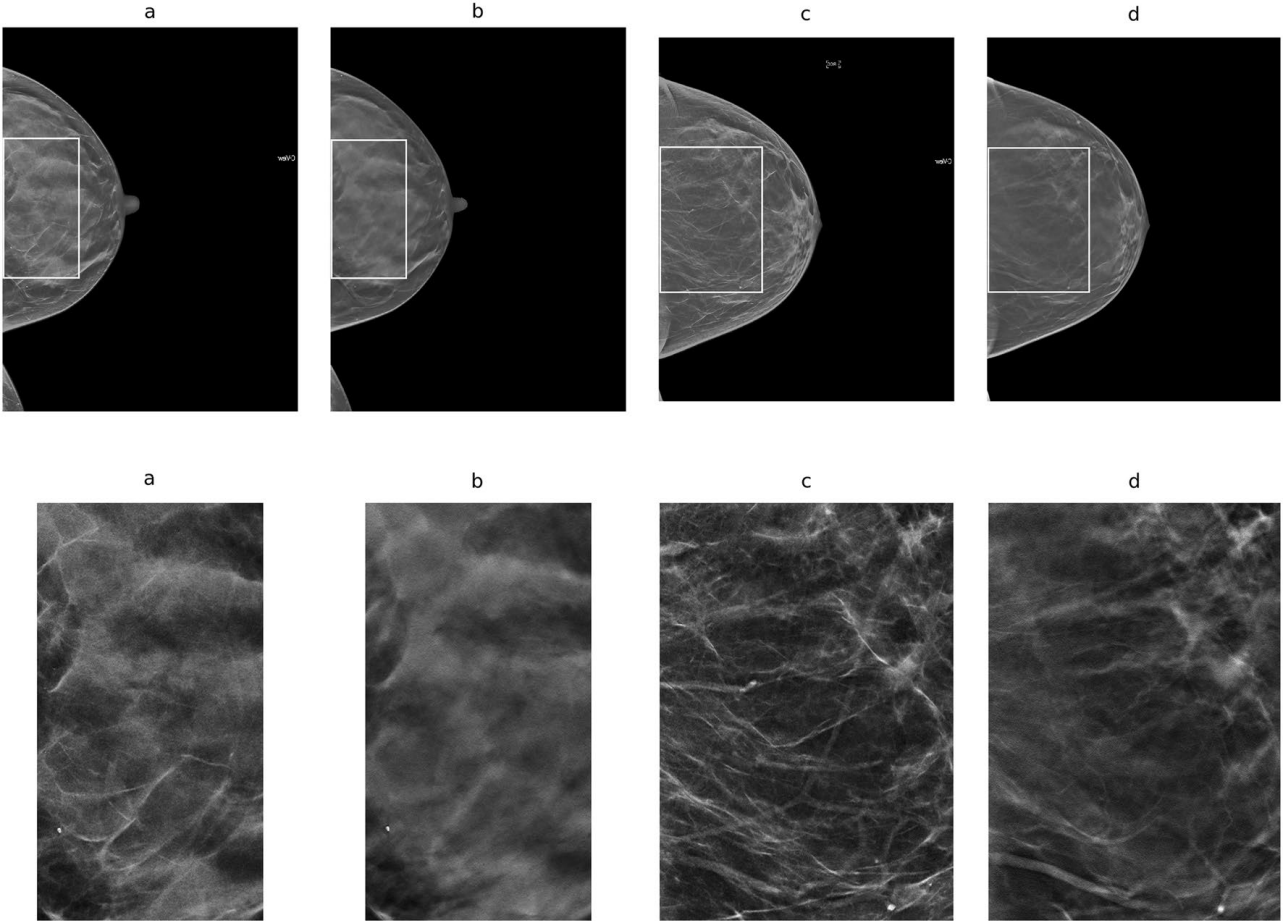
Image Illustrations: in the top row (**a**) C-View image for illustration-1; (**b**) respective central slice image; (**c**) C-View image for illustration-2; and (**d**) respective central slice image. Outlines in the top-row images are the largest rectangles that fit within the breast areas. These regions are shown in the second row with the same ordering for illustration purposes. Illustration-1 has 89 μm pixel spacing and illustration-2 has 107 μm pixel spacing.

**Figure 2:**
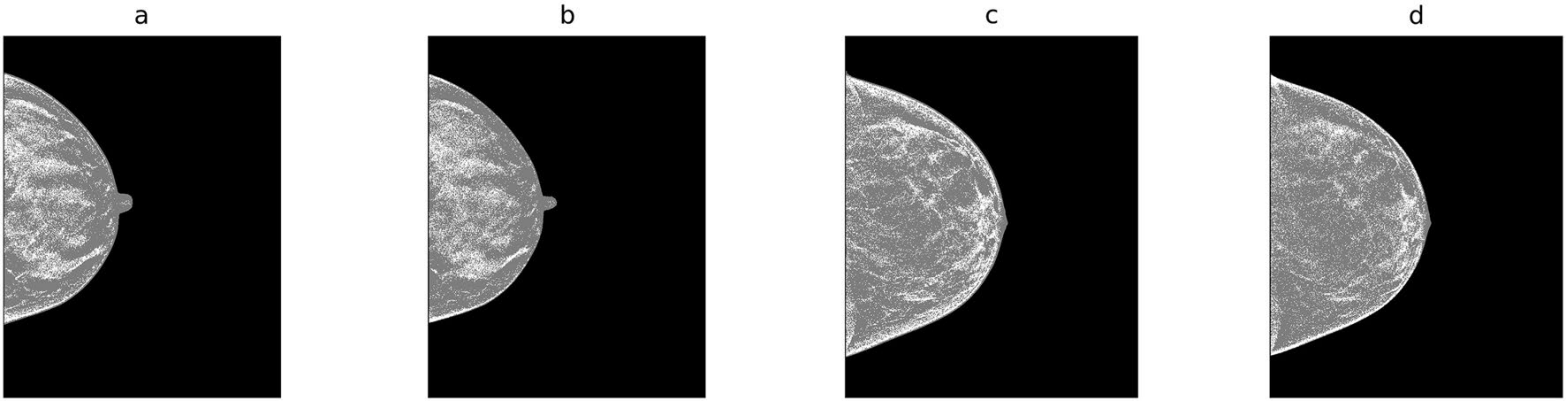
Breast Density Detection: this shows the density detection for the illustrations: (**a**) C-view, illustration-1; (**b**) respective central slice image; (**c**) C-view, illustration-2; and (**d**) respective central slice.

**Figure 3:**
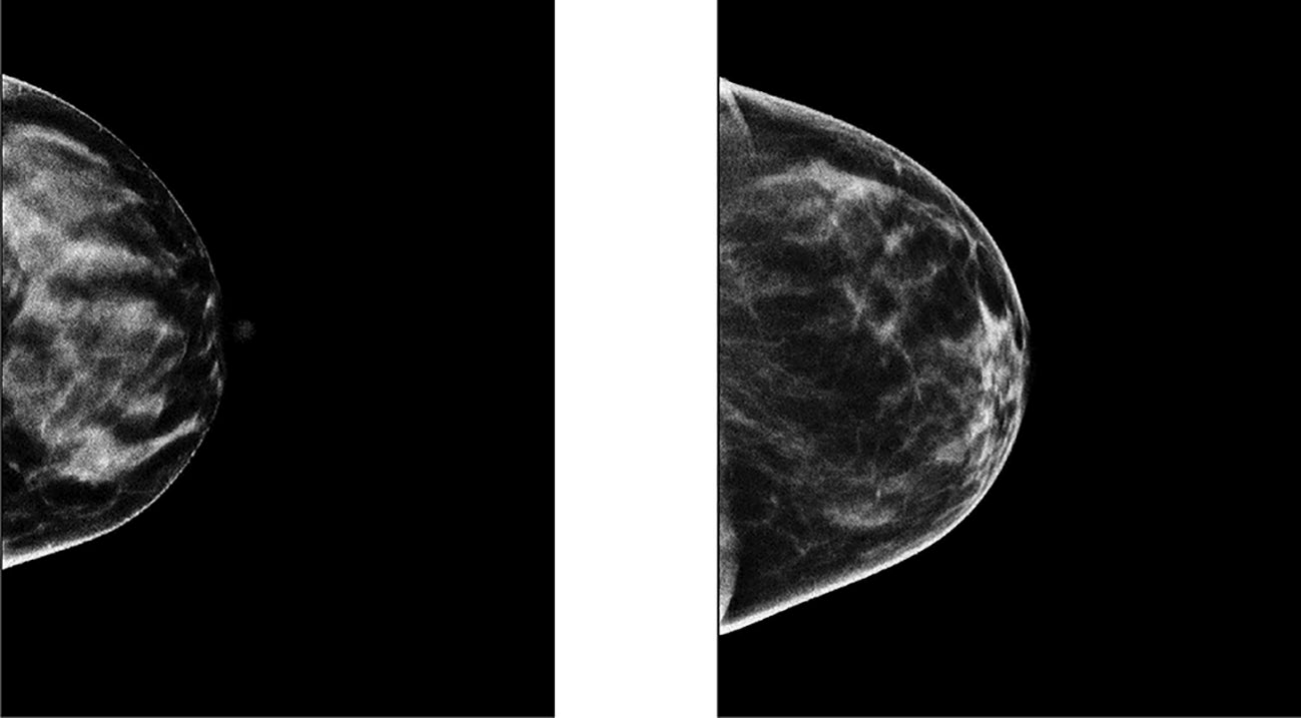
Projected Standardized Synthetic Breast Density Images: these show the standardized, s(x,y), images for illustration-1 (left) and illustration-2 (right) resulting from Eq. (8). Pixel values represent the percentage of dense tissue in the breast volume above their locations.

Several illustrations are used to show image data and the detection algorithm’s output*.* Figure 1 (top row) shows images from the two participants selected at random from left to right: (a) C-View image for illustration-1; (b) central DBT slice for illustration-1; (c) C-View image for illustration-2; and (d) central DBT slice for illustration-2. Illustration-1 has 89 μm pixel spacing and illustration-2 has 107 μm pixel spacing. The largest rectangle that fits within the breast area^34^ is outlined in each image. These regions are used for improved viewing purposes and are shown in the second row of Fig. 1. Although the breast structure in the volume slices track that of the C-View images, it appears less resolved. The density-detected images are shown in Fig. 2. These show the density labeling in the C-view images also tracks that of the labeling in the central volume slices. Figure 3 shows the synthetic s(x,y), images produced by the PD slice projections for these illustrations (from their labeled volumes). These appear as processed images (*for presentation*) but with level contrast as they are 8-bit images with limited dynamic range. These images illustrate another technique for creating synthesized 2D images in comparison with the C-View type images.

### Measurement modeling

**Figure 4:**
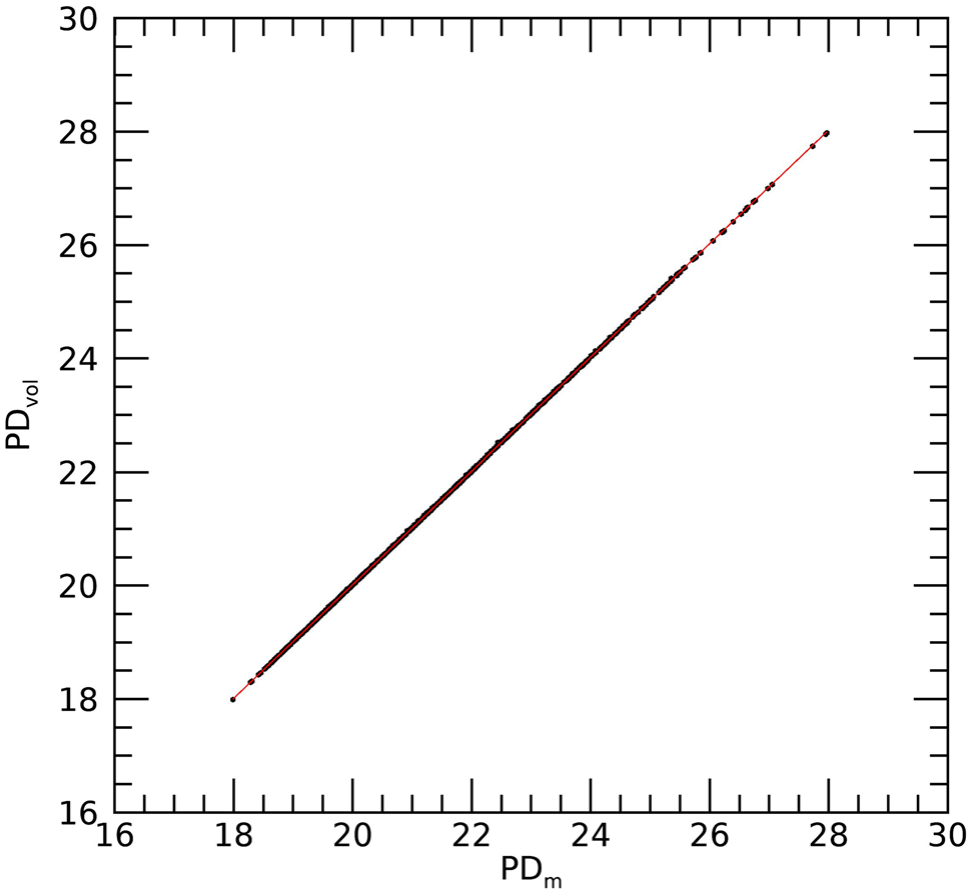
Regression analysis with PD_vol_ as a function of PD_m_ This shows the scatter plot between the two measures (points) and regression line (solid red), The analysis gave: slope ≈ 1.002, intercept ≈ − 0.0201, and linear correlation ≈ 1.0. The [mean, standard deviations] for the distributions were [21.60, 1.98] for PD_vol_ and [21.58, 1.98] for PD_m_.

**Figure 5:**
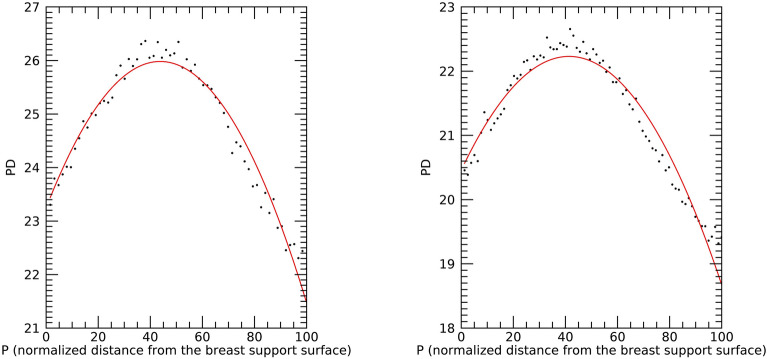
PD slice Profiles: this shows PD (y-axis) by slice number using the normalized distance from the breast support surface (P on the x-axis) for illustration-1 (left) and illustration-2 (right). PD values per slice number (points) were fitted with a second-degree polynomial (solid). The slice distance increases as the distance increases from the breast support surface (left side of each plot).

**Figure 6:**
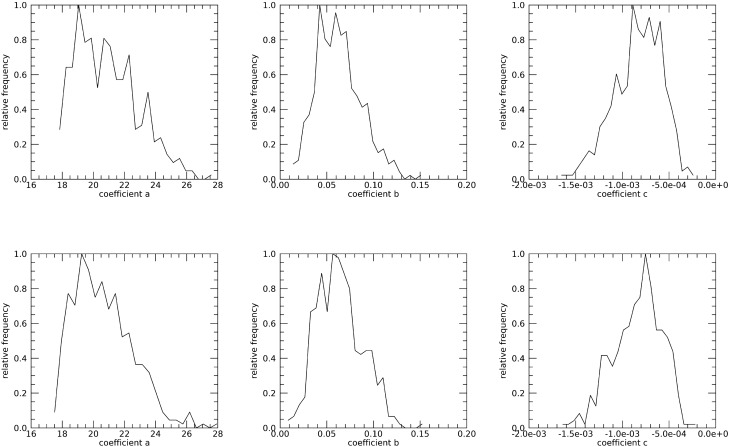
DBT Volume Slice 2nd Degree Polynomial Coefficient Histograms: these show the normalized histograms for the fit-coefficients, (a,b,c), separated by case (top-row) and control status (bottom-row).

Equations (5)-(8) show PD_vol_ and PD_m_ should be equivalent under the breast area similarity approximation. Figure 4 shows the scatter plots of these two measures (points) and the fitted regression line (solid red) with R ≈ 1.0, slope ≈ 1.002, and intercept ≈ − 0.0201 indicating the two measures are essentially *identical*. These findings show that both Eq. (8) and the interpretation of the synthesized images shown in Fig. 3 are valid. DBT slice modeling using Eq. (9) is shown in Fig. 5. There is also a cluster of PD quantities similar to the maximum in close slice proximity in both examples. Figure 6 shows the histograms for the Eq. (9) coefficients separated by case–control status. Averaging like coefficients for cases and controls gave: (a, b, c)_mean_ ≈ (21.1, 0.06, − 0.008) and (a, b, c)_mean_ ≈ (20.8, 0.07, − 0.0008), respectively. Applying a t-test across groups showed only the intercept (i.e., a) varied marginally (*p* value ≈ 0.046), as expected. Using parameter-means, the position with the greatest PD finding from Eq. (10) was approximately P ≈ 42 for either group. Empirically the mean maximum was P ≈ 40.7 with a standard error ≈ ± 0.28, showing close agreement with the model. Considering these findings, we investigated the maximum PD value as another measure.

### Breast cancer risk associations

Breast cancer associations are shown in Table 2. Both PD_syn_, [OR = 1.44 (1.18, 1.75)] and PD_vol_, [OR = 1.43 (1.18, 1.72)] were significant. ORs from PD_m_ were identical to PD_vol_. PD from the central slice was significant [OR = 1.42 (1.17, 1.72)] and similar to the slice with the largest PD quantity [OR = 1.47 (1.21, 1.78)]. The mean of DBT volume pixels (m_vol_) was also significant [OR = 1.31 (1.09, 1.57)] and similar to m_syn_ [OR = 1.29 (1.10, 1.52)]. Neither the total dense volume (D_v_) or the standard deviation within the volume (v_vol_) produced significant associations. We also compared PD from P = 1 (closest to breast support surface) and P = 100 (compression paddle) [not shown]; these provided significant breast cancer associations that were similar to either m_syn_ or m_vol_. When comparing the unadjusted to adjusted models in Table 2, the ORs for the BD measurements shifted considerably. Therefore, we investigated the correlation between BMI and the three main findings by first removing BMI outliers giving: R = [PD_syn_, PD_vol_, PD_m_] = [− 0.43, − 0.33, − 0.33]. Although this range of correlation is weak, it explains the confounding influence of BMI on BD measurements; the risk of breast cancer increases as either BD or BMI increases while these two factors move in opposition.

**Table 2:** Conditional Logistic Regression Modeling Results: this gives odd ratios (ORs) with 95% confidence intervals (CIs) parenthetically for each model; image measures were log-transformed.

n = 426 (pair)	SD	Unadjusted OR (95% CI) and Az (95% CI)	BMI and ethnicity adjusted OR (95% CI) and Az (95% CI)
PD_syn_			
OR	0.08	1.08 (0.92, 1.26)	1.44 (1.18, 1.75)
Az		0.51 (0.47, 0.56)	0.65 (0.61, 0.70)
D_v_			
OR	0.5513	1.23 (1.07, 1.42)	1.00 (0.82, 1.22)
Az		0.55 (0.51, 0.60)	0.63 (0.58, 0.67)
PD_vol_			
OR	0.0902	1.14 (0.97, 1.33)	1.43 (1.18, 1.72)
Az		0.53 (0.48, 0.57)	0.64 (0.60, 0.69)
PD_m_			
OR	0.0902	1.14 (0.97, 1.33)	1.43 (1.18, 1.72)
Az		0.52 (0.48, 0.57)	0.65 (0.60, 0.69)
m_vol_			
OR	0.00878	1.26 (1.06, 1.50)	1.31 (1.09, 1.57)
Az		0.55 (0.50, 0.59)	0.63 (0.59, 0.68)
v_vol_			
OR	0.5582	1.13 (0.89, 1.42)	1.19 (0.93, 1.53)
Az		0.51 (0.46, 0.56)	0.64 (0.60, 0.69)
m_syn_			
OR	1.0168	1.23 (1.05, 1.43)	1.29 (1.10, 1.52)
Az		0.56 (0.51, 0.60)	0.65 (0.60, 0.69)
PD (central slice)			
OR	0.0923	1.11 (0.95, 1.30)	1.42 (1.17, 1.72)
Az		0.52 (0.48, 0.57)	0.66 (0.61, 0.70)
PD (maximum slice)			
OR	0.0932	1.13 (0.97, 1.33)	1.47 (1.21, 1.78)
Az		0.53 (0.48, 0.57)	0.66 (0.61, 0.70)

Standard deviations (SDs) are provided for log-transformed distributions. The area under the receiver operating characteristic curve (Az) is given for each model with CIs parenthetically. From top to bottom: PD_syn_ is from C-View image analysis; D_v_ is the dense volume (mm^3^); PD_v_ is normalized volumetric PD; PD_m_ is the slice-mean; m_vol_ is the mean of the pixel values within the DBT volume (no processing); v_vol_ is the standard deviation of the pixel values within the DBT volume (no processing); and m_syn_ is the mean of the C-view image pixel values (no processing); PD from the central slice in the DBT volume at P ≈ 50; and PD (maximum slice) determined from the DBT slice with the largest PD finding.

**Figure 7:**
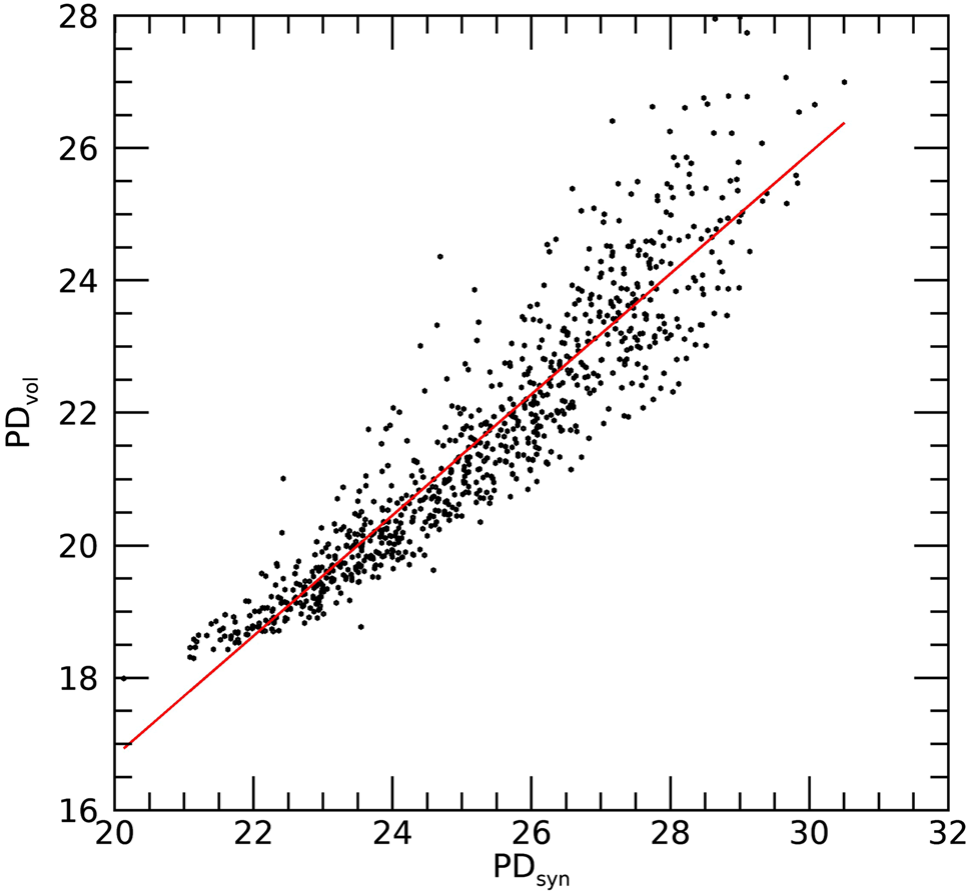
Regression analysis with PD_vol_ as a function of PD_syn_: this shows the scatter plot between the two measures (points) and regression line (red-solid): slope ≈ 0.92 and standard error ≈ 0.01, intercept ≈ − 1.4, and R ≈ 0.93. The [mean, standard deviations] for the distributions were [21.60, 1.98] for PD_vol_ and [25.25, 2.01] for PD_syn_.

The OR findings above were similar for PD_vol_ and PD_syn_. For risk factor purposes, this implies analyzing the C-View images (i.e., the breast volume structure projected onto a plane with heavy processing) is not subordinate to analyzing the volume images. To study these measures further, we investigated their relationship with linear regression. Figure 7 shows the scatter plot (points) and regression analysis (solid line): slope ≈ 0.92, intercept ≈ − 1.4, and R ≈ 0.93. Because the slope is close to unity, the intercept is not far from PD_vol_ = 0, and the strong positive correlation, these measures are approximately on the same scale and similar, although the plot does show nonlinear variation. Although the variation between these two measures increases as the respective measures increase, these regression findings assist in explaining the OR similarities found above.

## Discussion

The study demonstrated the validity of the density detection algorithm’s capability of translating to DBT by producing significant ORs. The volumetric measure was equivalent to the average PD values taken across the DBT slice images and agreed with the derivation showing these measures are approximately equivalent. Three other findings were notable as well: (1) the DBT slice with the largest PD finding was offset considerably from the central slice; (2) PD from the central slice, from the slice with maximum PD finding, or from the C-View image provided ORs similar to those from volumetric measure, and (3) the mean of the pixel values from the DBT volume slices or from the C-View images produced significant ORs without applying the detection processing. Where applicable, reference will be made to each of these three other notable findings in the following paragraphs with their respective finding number.

The PD slice profiling analysis is another novel aspect of this report. The related plots (Fig. 5) showed clusters of points (around 5–10 slices) with values close to the maximum PD value occurring around the curvature crest indicating why these isolated slices (finding number 2) provided similar ORs. We believe this is the first study to represent PD in this slice profile (derived from images that represent a volumetric rendering of the breast from x-ray technology). Other work that compared various PD-type measures (using FFDM) with a commercial volumetric breast density product that operates on 2D raw mammograms did not find large differences in ORs across the measures^40^. Our work agrees with these findings.

Breast cancer ORs between the volume and the synthetic images were almost identical; this indicates there is no benefit derived from analyzing the volume directedly, admitting the C-View image is derived from the volume. This finding applies to our method specifically but agrees with volumetric measures derived from 2D FFDM as follows. Comparisons with other techniques are often not exact due to study design variations such as sample size and model differences. Likewise, there is not an accepted convention for the standard deviation increment in the image measurement, which is distribution dependent for each image measure. However, the ORs for PD found in the report parallel results in other work to varying degrees: (1) agrees with those determined in these reports^24,38^; (2) are similar to those determined with volumetric measures^41^ (derived from conventional 2D mammograms); and (3) and are marginally less than a volumetric measure applied to DBT^28^, and it is worth noting that this DBT approach first operates on the 2D projection images, then applies machine learning to the reconstructed volume, and as evaluated had relatively few cancer observations. Finding number 3 above follows intuition as larger pixel values represent elevated levels of dense breast tissue. In the past, we have found the variation in conventional 2D mammograms provided significant ORs^41–43^, which did not hold in this study for the volume images. In this current study, D_v_ was not a significant risk factor but is the critical factor in the other measures. A significant OR was produced when normalizing D_v_ by the total breast volume, which supports the probability conjecture. Thus, the study provides insight into the nature of the traditional PD measurement (applied to 2D mammograms) and produced a related prescription for constructing a standardized synthesized 2D image.

Our findings can be summarized into two areas of investigation: (1) PD measurement development and validation; and (2) the nature of the anatomical volumetric distribution of PD (ORs and slice modeling), which may have biological importance (unknown at this time). We believe both areas represent new findings. As for measurement development, there are no universally accepted measures of breast density for 2D (let alone DBT) for multivariate risk prediction models in general. However, there are trends in this direction clinically. The standard measure for breast density in the US used for clinical reporting is the BI-RADS ordinal composition classification provided by the attending radiologist, originally developed for masking, or indicating when mammography may be ineffective. This measure is also used for risk prediction in both the BCSC^13,14^ and Tyrer-Cuzick models. The Tyrer-Cuzick model (and other models including the BCSC and Claus models) is also available in a widely used commercial mammography reporting software product (https://magview.com/risk-assessment/) to identify high-risk women within the radiology workflow. For DBT, the ACR recommends making the BI-RADS tissue assessments from either the synthetic 2D images (i.e., C-View images in this study) or the accompanying 2D FFDM images (supplement to BI-RADS lexicon 5th edition, 2013). It is also worth noting, in this 5th edition the tissue composition categories changed by dropping the quantitative component of the reporting due to reproducibility problems with volumetric measurements^44^. At this point, is not clear if a conventional measurement of BD, such as PD (studied here) translated to DBT, or if more involved methods derived from artificial intelligence^45^ will provide benefits if incorporated into risk prediction models used for clinical purposes beyond that provided by the BI-RADS measure because it is one measurement among many factors. Mammography imaging technology shifts can occur rapidly (discussed below) and are naturally ahead of the automated breast density measurement advancements. We could also posit the possibility that the available information within a mammogram of any type related to risk is somewhat limited and measurement reproducibility over longer timeframes is a critical measurement attribute for clinical applications.

There are several comments worth noting about this study. Absolute ground truth for volumetric breast density for an arbitrary breast was not known. Ideally, comparing our findings with breast phantoms designed for DBT with known mixtures of adipose and fibro-glandular tissue would be beneficial. In 2D (FFDM raw images), pixel values approximate the attenuated signal. Unfortunately, this characteristic is not preserved in the volume slices or 2D synthetic images due to the processing required for their construction. In any event, such phantoms would require realistic breast tissue spatial distributions for our approach to operate optimally due to its localized detection characteristic; this would preclude using more uniform type phantoms. To the best of our knowledge, the development of realistic anthropomorphic breast phantoms for DBT is a challenging problem and open area of research^46,47^. We have shown agreement (R > 0.7) between our PD measure and a calibrated phantom approach with FFDM images, where pixel values were mapped to standardized values^36^. We also found similar correlations when making comparisons with the operated-assisted PD method applied to both digitized film^30^ and FFDM^29^ images. Although not on the same scale, the monotonic relationship that our measure has with these other measurements is likely an essential attribute responsible for its risk prediction replication characteristic. Additionally, we analyzed a hospital-based population, where matching was used to account for case–control differences. Both the OR findings and summaries from Table 1 indicate this did not materially influence the outcomes. PD derivations are general and apply to any like metric, whereas the findings in this report apply specifically to our automated approach. We only analyzed cranial caudal mammograms, indicating we may have missed density information from the mediolateral views. Although the results indicated that 2D and 3D measures from PD were similar, the study design establishes a template that could be used for investigating other measures such as texture. Study images were from one type of DBT technology. It is worth noting, DBT technology is also shifting. For instance, the manufacturer of the units used for this study is now offering artificial intelligence enhanced images for DBT, smaller pixel spacing, and interleaved slice spacing (increased). The noise field multiplication modification analyzed here offers potential to apply to images derived from evolving DBT advances. The results from this study will require verification in other populations and DBT technologies as well.

## Data Availability

Mammography data can be obtained upon request to the corresponding author (JH, john.heine@moffitt.org).
